# Data on the financial performance of companies on BIST Sustainability 25 Index: An Entropy-based TOPSIS approach

**DOI:** 10.1016/j.dib.2024.110959

**Published:** 2024-09-19

**Authors:** İlker Kefe, Samet Evci, İrem Kefe

**Affiliations:** a Faculty of Economics and Administrative Sciences, Osmaniye Korkut Ata University, Osmaniye, Türkiye; b Faculty of Health Sciences, Osmaniye Korkut Ata University, Osmaniye, Türkiye

**Keywords:** BIST Sustainability 25 Index, Entropy-based TOPSIS model, Cash flow ratio, Financial statement analysis

## Abstract

In this data article, the Entropy-based TOPSIS technique is employed to assess the cash flow-based financial performance of companies. The study encompasses data from companies listed on the Borsa Istanbul (İstanbul Stock Exchange) and included in the BIST Sustainability 25 Index between 2018 and 2022. The performance metrics considered in the dataset are grouped into categories including liquidity, operational efficiency, financial structure, and profitability ratios. The dataset is derived from company balance sheets, income statements, and cash flow statements.

Specifications Table

**Table:** 

Subject	*Accounting, Finance*
Specific subject area	*Multi-criteria decision making, Entropy-based TOPSIS model.*
Data format	*Raw, Filtered, Analyzed.*
Type of data	*Excel file*
Data collection	*The dataset is collected from the financial statements of listed companies. The data is obtained from financial reports published on Borsa Istanbul (BIST) and the independent audit reports of the companies.*
Data source location	*A dataset containing publicly listed companies, excluding banks, included in the BIST Sustainability 25 Index between 2018 and 2022.*
Data accessibility	Repository name: Mendeley Data Data identification number: 10.17632/c6rwfjj5bt.2 Direct URL to data: https://data.mendeley.com/datasets/c6rwfjj5bt/2

## 1 Value of the Data

• The dataset provides financial insights into non-banking companies listed in the BIST Sustainability 25 Index (2018–2022), focusing on cash flow data alongside key financial statements.
• The dataset enables performance evaluation using liquidity, activity, financial structure, and profitability ratios, offering a comprehensive view of financial health.
• The dataset supports reproducibility, allowing for structured analysis using the Entropy and TOPSIS methods to rank companies’ financial performance.
• The dataset allows for sector-specific analysis, showing how financial performance changes over time across different industries.
• The dataset includes financial statement data to calculate both traditional ratios and cash flow ratios. Therefore, the dataset includes financial ratios that will allow for measuring companies' financial performance using different economic models.

## 2 Background

In recent years, sustainability has emerged as a critical focus for companies, influencing both financial strategies and performance outcomes [1]. The BIST Sustainability 25 Index provides a valuable opportunity to examine the intersection of sustainability practices and financial performance. Companies in this index are selected based on their commitment to sustainability, and understanding their financial health can offer insights into the long-term viability of sustainable business models.

Traditional financial ratios are used to estimate the performance of companies [2]. It is noteworthy that cash flow ratios are also used as a means of clarifying the findings of traditional ratios [3,4]. Cash flow ratios, when used in conjunction with other financial statement data, allow users to assess a company's liquidity and debt repayment capacity [5]. Traditional financial ratios, though widely used, may not fully capture the nuances of sustainable companies. Cash flow ratios, on the other hand, provide a clearer picture of liquidity and financial stability [6]. This combination provides a basis for developing various models to evaluate company operations, predict future performance, and compare companies [5]. The results suggest that cash flow ratios occasionally offer a more precise and accurate portrayal of the companies [7,8]. Moreover, due to their widespread usage and acceptance, cash flow ratios are favoured over traditional ratios [9]. This study leverages an Entropy-based TOPSIS approach to assess the financial performance of BIST Sustainability 25 companies, focusing on cash flow metrics over a five-year period. By doing so, the research contributes to the growing body of literature on sustainable finance, offering a comprehensive evaluation that accounts for both industry-specific and cross-sectoral variations in financial performance.

## 3 Data Description

The dataset offers insights into the financial state of BIST companies included in the Sustainability index. It enables researchers to contribute to performance research by using cash flow statement data in addition to basic financial statements such as balance sheets and income statements. This study focuses on the data of companies listed on BIST that operate outside the banking sector. The samples meet the following criteria:

• Companies must have complete data for all variables, including cash flow statements, income statements, and balance sheets.
• The banking sector has been excluded due to differing reporting standards.

In the literature, studies examining multiple periods often involve researchers evaluating financial performance based on individual periods or creating a single dataset to represent the examined period for their analyses. Studies with periods longer than 5 years include López et al. [10], Sudha [11], and Arias Fogliano de Souza Cunha and Samanez [12], while those with periods shorter than 5 years include Pérez-Calderón et al. [13] and Oberndorfer et al. [14]. In addition, de Castro Sobrosa Neto et al. [15], Stekelenburg et al. [16], Karakaya and Kutlu [17], and Levent [18] use a 5-year time frame in their sustainability index studies. The data collection period from 2018-2022 is chosen to capture a complete economic cycle. This time frame allows for a more comprehensive analysis of financial health across different economic conditions. Therefore, data from a 5-year period, including the year 2022, are incorporated into the analysis. This study employs annual Entropy analysis results to develop a new decision matrix with years as the criteria set. This matrix is then weighted using the Entropy method, analyzed with the TOPSIS method, and financial performance rankings of the companies are obtained. The study data includes companies listed in the BIST Sustainability 25 Index. This inclusion provides a holistic view of financial performance by incorporating companies from various sectors within the index, aiming to present a more inclusive and representative analysis of financial health and sustainability practices. The dataset includes 21 companies listed in the Sustainability index, which were analyzed using the Entropy and TOPSIS (Technique of Order Preference Similarity to the Ideal Solution) methods. Table 1 shows the companies included in the BIST Sustainability index (XSD25).

**Table 1:** Companies in the BIST Sustainability Index.

Constituent Code-Company Name	Company Code	Operating Sector
ARCLK.E-ARÇELİK A.Ş.	C1	Consumer Electronics
BIMAS.E-BİM BİRLEŞİK MAĞAZALAR A.Ş.	C2	Retail
CIMSA.E-ÇİMSA ÇİMENTO SANAYİ VE TİCARET A.Ş.	C3	Cement and Construction Materials
DOAS.E-DOĞUŞ OTOMOTİV SERVİS VE TİCARET A.Ş.	C4	Automotive
DOHOL.E-DOĞAN ŞİRKETLER GRUBU HOLDİNG A.Ş.	C5	Holding (Diversified Investments)
ENJSA.E-ENERJİSA ENERJİ A.Ş.	C6	Energy
ENKAI.E-ENKA İNŞAAT VE SANAYİ A.Ş.	C7	Construction and Engineering
EREGL.E-EREĞLİ DEMİR VE ÇELİK FABRİKALARI T.A.Ş.	C8	Steel and Iron
FROTO.E-FORD OTOMOTİV SANAYİ A.Ş.	C9	Automotive
KCHOL.E-KOÇ HOLDİNG A.Ş.	C10	Holding (Diversified Investments)
MGROS.E-MİGROS TİCARET A.Ş.	C11	Retail
PETKM.E-PETKİM PETROKİMYA HOLDİNG A.Ş.	C12	Petrochemicals
PGSUS.E-PEGASUS HAVA TAŞIMACILIĞI A.Ş.	C13	Airlines
SAHOL.E-HACI ÖMER SABANCI HOLDİNG A.Ş.	C14	Holding (Diversified Investments)
SISE.E-TÜRKİYE ŞİŞE VE CAM FABRİKALARI A.Ş.	C15	Glass and Packaging
TAVHL.E-TAV HAVALİMANLARI HOLDİNG A.Ş.	C16	Airport Operations
TCELL.E-TURKCELL İLETİŞİM HİZMETLERİ A.Ş.	C17	Telecommunications
THYAO.E-TÜRK HAVA YOLLARI A.O.	C18	Airlines
TOASO.E-TOFAŞ TÜRK OTOMOBİL FABRİKASI A.Ş.	C19	Automotive
TTRAK.E-TÜRK TRAKTÖR VE ZİRAAT MAKİNELERİ A.Ş.	C20	Agricultural Machinery
VESTL.E-VESTEL ELEKTRONİK SANAYİ VE TİCARET A.Ş.	C21	Consumer Electronics

The sectors analysed in this study can be broadly classified into high-tech and low-tech categories to enhance the generalizability of the findings. High-tech sectors, such as consumer electronics, telecommunications, automotive, and airlines, are characterized by rapid technological advancements and innovation-driven financial performance. In contrast, low-tech sectors, including retail, cement and construction materials, energy, and glass and packaging, exhibit more stable, incremental financial trends. By categorizing the sectors in this manner, the study captures a diverse range of financial behaviors, allowing for a more representative analysis of company performance and sustainability practices. This classification elevates the generalizability of the findings by demonstrating how sectoral variations influence financial health and sustainability outcomes across different industries. The sampling method involves selecting companies listed on the BIST Sustainability 25 Index, which comprises companies with high sustainability performance. To qualify for this index, companies must score 70 or above in the General Sustainability Rating, 60 or above in each main category, and at least 8 category scores of 50 or above. The BIST Sustainability 25 Index includes companies from the Star Market, Main Market, and Sub-Market that volunteer for the sustainability assessment. For this study, companies are chosen based on their continuous listing on the BIST Sustainability 25 Index and their availability of complete financial data (balance sheets, income statements, and cash flow statements) for the period 2018-2022. Companies are also selected based on their high trading volume and market value among those meeting these sustainability criteria. The selection of the 21 companies included in this study reflects these criteria.

The dataset is collected from the financial statements of listed companies. Financial data of the companies within the scope of the study are obtained by using financial reports published on BIST, official websites of the companies and independent audit reports. The data collection protocol involves obtaining financial statements directly from the respective companies' annual reports and verified financial databases. This approach ensures the reliability and accuracy of the data. The sampling method is designed to include companies from various industries to provide a diverse and representative sample. The 21 companies are selected based on their availability of complete financial data for the entire period and their significance in their respective industries.

With the integration of the cash flow statement into financial statements, additional information has become accessible for financial assessments [19]. Considering the criteria of usage and compatibility in similar relevant research [[2], [3], [4],[20], [21], [22], [23], [24]], a total of 12 cash flow ratios were selected by categorizing liquidity ratios, operating ratios, financial structure ratios, and profitability ratios. The cash flow rates used as comparison metrics in the analysis are derived from companies' balance sheet, income statement, and cash flow statement data.

Table 2 presents the indicators based on the cash flow statement, encompassing liquidity ratios, operating ratios, financial structure ratios, and profitability ratios. The raw data file for the companies included in the study can be accessed at https://data.mendeley.com/datasets/c6rwfjj5bt/2. This data file comprises ratios for the years 2018-2022, including dividends paid, trade receivables, non-current assets, total assets, current liabilities, non-current liabilities, total liabilities, total equity, revenue, financial expense, cash flow from operating activities, and cash flow from financing activities.

**Table 2:** Cash flow ratios used.

Ratio Group	Code Ratio Used	Meaning
Liquidity Ratios	CFR1 **Cash-STL Ratio =** CFO / STL	Measures the ability of a company to cover its short-term liabilities with cash flow from operations.
	CFR2 **Critical Needs Ratio =** CFO / (I + STL + DP)	Assesses the company's ability to meet critical financial obligations, including interest payments, short-term liabilities, and dividends paid, with its operating cash flow.
	CFR3 **Cash Ratio =** (CFO – DP) / STL	Indicates the ability to cover short-term liabilities with cash flow from operations after accounting for dividends paid.

Operating Ratios	CFR4 **Cash-Asset Ratio** = CFO / TA	Shows the proportion of total assets financed by cash flow from operations.
	CFR5 **Cash-Fixed Asset Ratio** = CFO / FA	Measures the ability to generate cash flow from operations relative to fixed assets.
	CFR6 **Cash-Trade Receivables Ratio** = CFO / TR	Assesses the efficiency of converting trade receivables into cash flow from operations.

Financial Structure Rates	CFR7 **Cash-Debt Ratio** = CFO / TL	Evaluates the company's capacity to pay off its total liabilities using cash flow from operations.
	CFR8 **External Financing Index Ratio** = CFO / CFFA	Determines the proportion of cash flow from operations used for external financing activities.

Profitability Ratios	CFR9 **Cash-Sales Ratio** = CFO / NS	Measures the cash flow from operations relative to net sales.
	CFR10 **Cash-Assets Ratio** = CFO / TA	Shows the proportion of total assets financed by cash flow from operations.
	CFR11 **Cash-Equity Ratio** = CFO / E	Indicates the amount of cash flow from operations relative to the company's equity.
	CFR12 **Cash-Working Capital Ratio** = CFO / (E + LTL)	Evaluates the company's ability to cover its working capital needs with cash flow from operations.

*Note:* CFO = Cash Flow from Operation, STL = Short-Term Liabilities, I = Interests, DP = Dividends Paid, TA = Total Assets, FA = Fixed Assets, TR = Trade Receivables, TL = Total Liabilities, CFFA = Cash Flow from Financing Activities, NS = Net Sales, E = Equity, LTL = Long-Term Liabilities.

Using the ratios in Table 2, the ratios of 21 companies in the sustainability index for the years 2018-2019-2020-2021 and 2022 were calculated and made suitable for Entropy and TOPSIS analysis.

## 4 Experimental Design, Materials and Methods

The criteria weights of the obtained cash flow ratios are identified using the Entropy method. First, a decision matrix is created. While creating the decision matrix, negative values are transformed using the Z-score standardization developed by Zhang et al. [25]. This process normalizes the decision matrix. Subsequently, the Entropy values (e_j_) related to the criteria are found, the degrees of diversification (d_j_) are calculated, and the Entropy criteria weights (w_j_) are determined. The criteria weights of the ratios for the years 2018-2022 are determined using cash flow ratios. Table 3 shows the criteria weights calculated based on cash flow ratios.

**Table 3:** Criterion weights calculated with the Entropy method.

		Liquidity Ratios			Operating Ratios			Financial Structure Rates		Profitability Ratios			
		CFR1	CFR2	CFR3	CFR4	CFR5	CFR6	CFR7	CFR8	CFR9	CFR10	CFR11	CFR12
2018	1/ln(m)	0,3285											
	e_j_	0,9739	0,9469	0,9670	0,9728	0,9737	0,9628	0,9785	0,9704	0,9770	0,9728	0,9183	0,9477
	d_j_	0,0261	0,0531	0,0330	0,0272	0,0263	0,0372	0,0215	0,0296	0,0230	0,0272	0,0817	0,0523
	w_j_	0,060	0,121	0,075	0,062	0,060	0,085	0,049	0,068	0,052	0,062	0,186	0,119
	Rank	10	2	5	7	9	4	12	6	11	7	1	3
2019	1/ln(m)	0,3285											
	e_j_	0,9007	0,9824	0,9010	0,9166	0,8953	0,7391	0,9224	0,9644	0,7665	0,9166	0,6925	0,8831
	d_j_	0,0993	0,0176	0,0990	0,0834	0,1047	0,2609	0,0776	0,0356	0,2335	0,0834	0,3075	0,1169
	w_j_	0,065	0,012	0,065	0,055	0,069	0,172	0,051	0,023	0,154	0,055	0,202	0,077
	Rank	6	12	7	8	5	2	10	11	3	8	1	4
2020	1/ln(m)	0,3285											
	e_j_	0,8748	0,9675	0,7872	0,8863	0,8168	0,8164	0,8941	0,5132	0,9402	0,8863	0,1748	0,8789
	d_j_	0,1252	0,0325	0,2128	0,1137	0,1832	0,1836	0,1059	0,4868	0,0598	0,1137	0,8252	0,1211
	w_j_	0,049	0,013	0,083	0,044	0,071	0,072	0,041	0,190	0,023	0,044	0,322	0,047
	Rank	6	12	3	8	5	4	10	2	11	8	1	7
2021	1/ln(m)	0,3285											
	e_j_	0,9124	0,9436	0,9205	0,9134	0,8793	0,7618	0,9213	0,9322	0,8490	0,9134	0,7392	0,9034
	d_j_	0,0876	0,0564	0,0795	0,0866	0,1207	0,2382	0,0787	0,0678	0,1510	0,0866	0,2608	0,0966
	w_j_	0,062	0,040	0,056	0,061	0,086	0,169	0,056	0,048	0,107	0,061	0,185	0,068
	Rank	6	12	9	7	4	2	10	11	3	7	1	5
2022	1/ln(m)	0,3285											
	e_j_	0,9158	0,8947	0,9230	0,9205	0,8396	0,7587	0,9287	0,7225	0,8874	0,9205	0,8685	0,8786
	d_j_	0,0842	0,1053	0,0770	0,0795	0,1604	0,2413	0,0713	0,2775	0,1126	0,0795	0,1315	0,1214
	w_j_	0,055	0,068	0,050	0,052	0,104	0,157	0,046	0,180	0,073	0,052	0,085	0,079
	Rank	8	7	11	9	3	2	12	1	6	9	4	5

These ratios and weights vary each year and are used in evaluating the performance of companies.

After the Entropy results are derived, the cash flow ratio data of 21 companies are analyzed using the TOPSIS method. The derived criteria weights are used in the TOPSIS method, and the companies are ranked based on their cash flow ratios. First, a decision matrix (A) is created using the companies' financial data. Then, a standardized decision matrix (R) is prepared by normalizing the decision matrix. In the next stage, a weighted decision matrix (V) is created, and the positive ideal (A*) and negative ideal (A^−^) solution values are determined. The distances to the positive (S_i_*) and negative (S_i_^−^) ideal points are calculated, and the relative closeness to the ideal solution (C_i_*) is found. Table 4 shows the TOPSIS results calculated based on cash flow ratios.

**Table 4:** TOPSIS ranking.

Companies	2018		2019		2020		2021		2022	
	C_i_*	Rank	C_i_*	Rank	C_i_*	Rank	C_i_*	Rank	C_i_*	Rank
C1	0,346	16	0,309	20	0,210	10	0,132	20	0,081	19
C2	0,457	7	0,393	8	0,246	8	0,252	5	0,233	11
C3	0,379	13	0,325	18	0,192	12	0,134	19	0,153	16
C4	0,196	21	0,337	17	0,172	14	0,169	16	0,106	18
C5	0,592	2	0,433	3	0,172	13	0,334	3	0,283	9
C6	0,359	14	0,376	11	0,242	9	0,213	11	0,277	10
C7	0,324	19	0,378	10	0,195	11	0,219	10	0,063	20
C8	0,417	8	0,426	5	0,278	7	0,161	17	0,171	14
C9	0,477	5	0,357	14	0,327	3	0,332	4	0,366	7
C10	0,348	15	0,204	21	0,138	18	0,201	13	0,333	8
C11	0,660	1	0,697	1	0,652	1	0,588	1	0,588	1
C12	0,487	3	0,362	13	0,327	4	0,128	21	0,134	17
C13	0,396	11	0,404	7	0,123	19	0,251	6	0,422	4
C14	0,199	20	0,380	9	0,115	21	0,235	7	0,192	12
C15	0,345	17	0,340	15	0,293	6	0,144	18	0,180	13
C16	0,462	6	0,319	19	0,117	20	0,171	15	0,160	15
C17	0,482	4	0,414	6	0,302	5	0,503	2	0,499	2
C18	0,403	9	0,339	16	0,146	17	0,220	9	0,374	6
C19	0,402	10	0,429	4	0,169	15	0,199	14	0,495	3
C20	0,331	18	0,493	2	0,360	2	0,224	8	0,385	5
C21	0,380	12	0,369	12	0,168	16	0,210	12	0,019	21

**Fig. 1:**
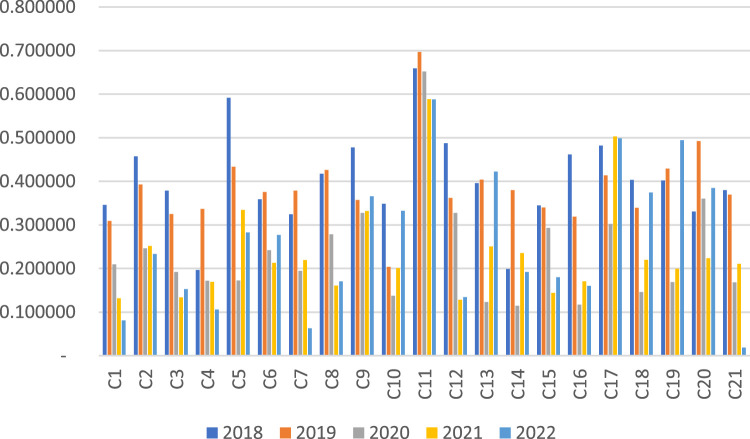
Financial performance scores of companies in the period 2018-2022.

To better observe the TOPSIS results, the data presented in Table 4 are illustrated in Fig. 1.

Fig. 1 illustrates the TOPSIS results for 21 companies (C1 to C21) over the years 2018 to 2022, based on their cash flow ratios. the chart enables a comparative assessment of companies' financial health and stability based on their cash flow performance evolution. Each bar represents the relative closeness (C_i_*) to the ideal solution for each company in a given year. As shown in Fig. 1, fluctuations in the cash flow performance of companies over a five-year period are depicted. Certain companies, like C11 (MİGROS TİCARET A.Ş.), exhibit consistent high closeness values, indicating stable cash flow ratios. Meanwhile, others, including C1 (ARÇELİK A.Ş.), C2 (BİM BİRLEŞİK MAĞAZALAR A.Ş.), and C5 (DOĞAN ŞİRKETLER GRUBU HOLDİNG A.Ş.), display notable ranking fluctuations. C11 attained the highest C_i_* value in 2019, suggesting its proximity to the ideal solution compared to peers.

## Limitations

The dataset is limited to a five-year period from 2018 to 2022. Due to the implementation of inflation adjustments in Türkiye in 2023 and 2024, the data set has been compiled up to the year 2022.

## Ethics Statement

The research did not involve any human subjects or animal experiments. Social media platforms weren't a source of data collection either.

## CRediT authorship contribution statement

**İlker Kefe:** Conceptualization, Methodology, Software, Investigation, Formal analysis, Writing – original draft. **Samet Evci:** Investigation, Formal analysis, Supervision, Writing – review & editing. **İrem Kefe:** Methodology, Investigation, Software, Writing – review & editing.

## Data Availability

Data on the financial performance of companies on BIST Sustainability 25 Index: An Entropy-based TOPSIS approach (Original data) (Mendeley Data).
